# Validated Growth Rate-Dependent Regulation of Lipid Metabolism in *Yarrowia lipolytica*

**DOI:** 10.3390/ijms23158517

**Published:** 2022-07-31

**Authors:** Naghmeh Poorinmohammad, Jing Fu, Bob Wabeke, Eduard J. Kerkhoven

**Affiliations:** 1 Department of Biology and Biological Engineering, Chalmers University of Technology, SE-412 96 Gothenburg, Sweden; nagpoo@chalmers.se (N.P.); jingf@chalmers.se (J.F.); bob_wabeke@hotmail.com (B.W.); 2 Novo Nordisk Foundation Center for Biosustainability, Chalmers University of Technology, SE-412 96 Gothenburg, Sweden

**Keywords:** lipid accumulation, *Yarrowia lipolytica*, comparative proteomics, oleaginous yeast

## Abstract

Given the strong potential of *Yarrowia lipolytica* to produce lipids for use as renewable fuels and oleochemicals, it is important to gain in-depth understanding of the molecular mechanism underlying its lipid accumulation. As cellular growth rate affects biomass lipid content, we performed a comparative proteomic analysis of *Y. lipolytica* grown in nitrogen-limited chemostat cultures at different dilution rates. After confirming the correlation between growth rate and lipid accumulation, we were able to identify various cellular functions and biological mechanisms involved in oleaginousness. Inspection of significantly up- and downregulated proteins revealed nonintuitive processes associated with lipid accumulation in this yeast. This included proteins related to endoplasmic reticulum (ER) stress, ER–plasma membrane tether proteins, and arginase. Genetic engineering of selected targets validated that some genes indeed affected lipid accumulation. They were able to increase lipid content and were complementary to other genetic engineering strategies to optimize lipid yield.

## 1. Introduction

*Yarrowia lipolytica* is an oleaginous yeast capable of accumulating high amounts of lipids under nitrogen-limitation conditions, primarily as triacylglycerols (TAGs). This arouses significant biotechnological interest, as TAGs can be used for biofuel production and intermediates for other oleochemicals. As a model unconventional oleaginous yeast that is genetically tractable and with available genomic information, *Y. lipolytica* is arguably among the best candidates for industrial lipid production [1]. However, to make production meet industrial scale requirements requires further improvement. For this, effective implementation of modern biotechnological approaches, such as metabolic engineering and synthetic biology, is instrumental, but a deeper understanding of the molecular mechanisms behind the oleaginous phenotype is paramount. Over the last few decades, lipid accumulation in different oleaginous microorganisms has been studied and is believed to be the consequence of slower growth in nutrient deficiency while having carbon source in excess [2]. Generally, the molecular specificities leading to this phenotype remain largely unclear.

Analyzing different types of omics-level data can efficiently improve the understanding via a holistic view on lipid accumulation mechanisms. In this regard, a few attempts have been successfully made to reveal the molecular mechanism behind lipid accumulation at the transcriptomic level in *Y. lipolytica*. This is while no comprehensive focus on the proteomics of lipid accumulation data analyses has yet been performed in this yeast. Accordingly, other than elucidation of the key enzymes that are directly involved in lipid biosynthesis, several other genes have been reported to be associated with lipid accumulation, as observed from transcriptome analyses [2]. More importantly, an aspect that not has been thoroughly investigated, however, are the observations that the levels of lipid accumulation correlate with growth rate in various oleaginous yeasts [3,4,5,6]. Generally, lower growth rates have been associated with higher lipid accumulations. Further investigation of this phenomenon would likely broaden our understanding of lipid accumulation biology in this yeast.

To this end, we undertook the first comparative proteomic study on a lipid over-accumulating mutant of *Y. lipolytica* at different growth rates. To differentiate the regulatory response related to lipid accumulation from the more general response to growth rate differences, we included a non-lipid accumulation reference strain. Upon identification of proteins involved in regulating the lipid-accumulating phenotype, we validated these results by genetic engineering of selected promising candidate genes.

It is believed that information gained from the data produced is required for improvement in our understanding of mechanisms involved in the transition of growth to lipid accumulation which will finally be helpful in identifying targets for metabolic or genetic engineering in *Y. lipolytica*.

## 2. Results and Discussion

### 2.1. Relationship between Growth and Lipid Accumulation in Y. lipolytica

Lipid metabolism and its basic biology have been well studied in *Y. lipolytica*, many of which are shared with other oleaginous yeasts. Lipid accumulation in other oleaginous yeasts has been shown to be growth rate-dependent and maximal lipid amounts to be accumulated at lower growth rates [3,4,5,6]. We postulated that a similar behavior would be observed in *Y. lipolytica*, and if so, this would render this phenomenon a promising approach to further elucidate the regulation of the lipid accumulation phenotype.

**Figure 1:**
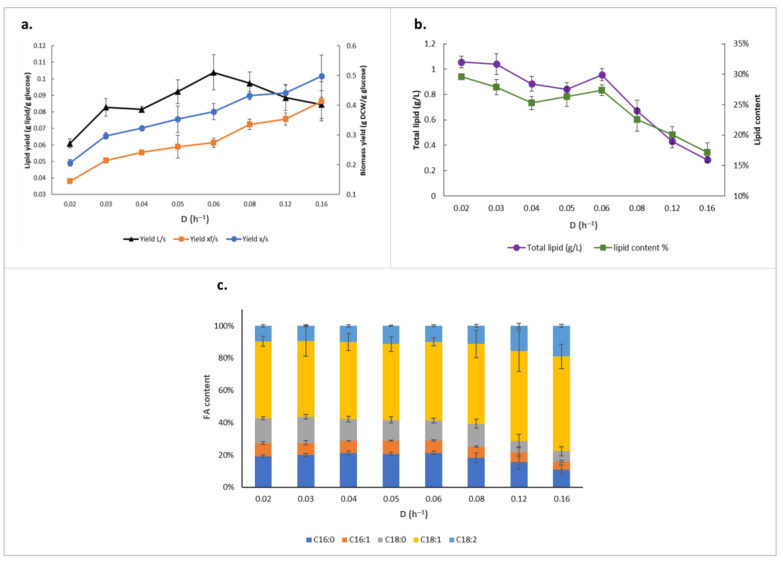
Lipid production profile of *Y. lipolytica* OKYL040 at different dilution rates. (**a**) Lipid and biomass yields (L: lipid, s: substrate which is glucose here, xf: non-lipid biomass, x: biomass); (**b**) total lipid production; (**c**) fatty acid (FA) composition.

To test this, we cultivated the *Y. lipolytica* OKYL049 strain, which has been genetically engineered for improved lipid accumulation [7], in nitrogen-limiting chemostats with dilution rates (D) ranging from 0.02 h^−1^ to 0.16 h^−1^ (Table 1). The maximum concentration of total lipids and lipid content was 1.06 g/L and 30%, respectively, at D = 0.02 h^−1^. However, while the nonlipid biomass yields on glucose showed a consistent increase over the tested dilution rate range, lipid yield on glucose demonstrates a nonlinear pattern, with lower yields at higher and lower dilution rates, while a maximum is reached at D = 0.06 h^−1^ (Figure 1a). This denotes that lipid production in *Y. lipolytica* also has a strong dependence on growth rate during nitrogen limitation, and that the yeast favors lipid biosynthesis at specific dilution rates. This represents a true redirection of intracellular fluxes, as the specific glucose uptake rate consistently increased at higher growth rates (Table 1), even while the lipid yield peaked at 0.104 g lipid/g glucose. Moreover, although total lipid concentration and lipid content is the highest while cells are grown at the lowest tested dilution rate, there seemed to be a shoulder peak at D = 0.06 h^−1^, breaking up the declining trend through higher dilution rates (Figure 1b). Micrographs after staining of *Y. lipolytica* OKYL049 lipid bodies illustrate the same pattern (Figure S1, Supplementary File S2).

In terms of fatty acid profile, other than a slight increase in C18:1 and C18:2 content in dilution rates of more than 0.12 h^−1^, no significant differences in the proportions were observed amongst cultivations at different dilution rates, with oleic acid and palmitic acid being the dominant fatty acids in all cultivations (Figure 1c).

To investigate the observed growth rate-dependent lipid accumulation via a proteomic approach, we defined a factorial experimental design and included a strain that is depleted of the ability to accumulate triacylglycerols. The so-called Q4 strain, deficient in the acyltransferases YlDGA1, YlDGA2, YlLRO1, and YlARE1, has previously been used to study the role of each acyltransferase independently [8,9,10]. The use of the Q4 strain as control in our analysis would allow us to differentiate the regulatory responses into those that are either (a) only due to differences in growth rate, or (b) observed if the difference in growth rate coincides with a change in lipid accumulation. As anticipated, the lipid content of the Q4 strain was not affected by the tested growth rates (Figure S2, Supplementary File S2), indicating the feasibility of our strategy. However, the Q4 strains had a lower maximum growth rate, such that cultivations with dilution rates over 0.1 h^−1^ resulted in washout from the chemostat. Accordingly, we settled on sampling from nitrogen-limited chemostat cultivations of the *Y. lipolytica* Q4 strain (from here on referred to as *control strain*) and *Y. lipolytica* OKYL049 strain at D = 0.06 h^−1^ (optimum for highest lipid yield) and D = 0.1 h^−1^ (nonoptimal for lipid production matrices).

### 2.2. Effect of Growth Rate on Proteome Composition of Y. lipolytica during Lipogenic Conditions

**Figure 2:**
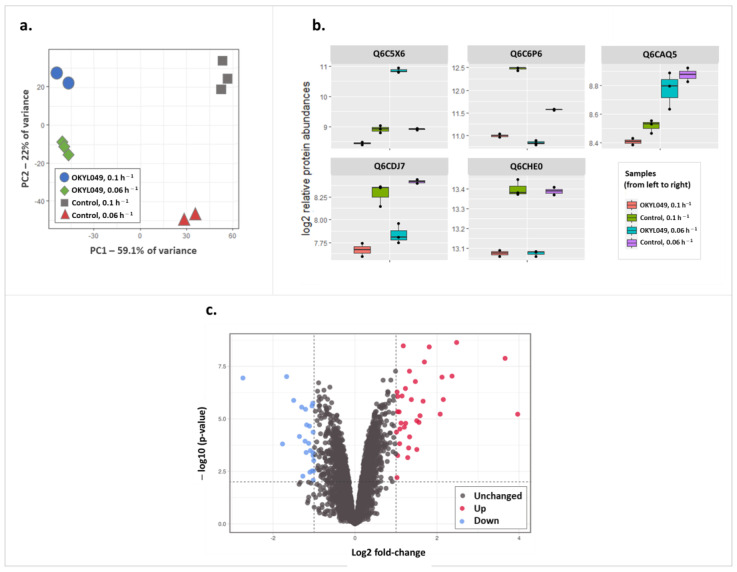
Differential expression analysis on the proteomic data. (**a**) PCA (principal component analysis) plot highlighting similarities between individual replicates and clear separation between test groups; (**b**) box plots of examples of protein expression-change behaviors that the current study is or is not interested in (see text); (**c**) volcano plot highlighting differentially expressed proteins in OKYL049 with lower dilution rate compared to the control strain. The dots in red and blue indicate proteins with significantly altered levels (|log2 FC| > 1, *p*-value < 0.01).

A combined number of 4441 proteins were detected from all samples, with no substantial difference across samples in the number of proteins identified (Supplementary File S3). Principal component analysis (PCA) was performed to ensure reproducibility across replicates. Accordingly, the first two PCs accounted for 81% of protein variance and showed a clear separation of all groups (Figure 2a). As shown in Figure 2a, OKYL049 samples were well separated from the control samples subjected to pressure by PC1, while PC2 seemed to better distinguish the dilution rates 0.1 and 0.06. Overall, PCA analysis reflects pronounced proteome changes in OKYL049 vs. the control strain in the two tested dilution rates.

To identify the differential protein expression patterns that concurred with the maximum lipid content at D = 0.06 h^−1^, we extracted the interaction term of a generalized linear model. As such, the results presented here describe how protein expression is affected when lipid yield is maximized. This is obtained by comparing protein expression at D = 0.06 h^−1^ relative to D = 0.10 h^−1^ (cf. Figure 1a), while simultaneously filtering out those protein expression changes that correlated with dilution rate, but not with the lipid content (due to the inability of the control strain to accumulate triacylglycerols). The remaining protein expression changes are therefore only correlating with the maximum lipid yield (Figure 2b). To exemplify, the expression profile of formate dehydrogenase (Q6C5X6, Figure 2b) showed no change in the control strain when comparing dilution rates, but its expression drastically increased at maximum lipid yield in the OKYL049 strain at 0.06 h^−1^. In addition, arginase (Q6C6P6, Figure 2b) was downregulated in both control and OKYL49 strains, signifying growth-correlated expression; however, the scale of the downregulation was far less profound in the lipid-accumulating strain in comparison to the control. Such expression profiles, where the OKYL049 strain and the control strain show quantitatively different responses to the growth rate, are identified when extracting the interaction term of the linear model. Contrastingly, the proteins Q6CAQ5 (E3 ubiquitin-protein ligase), Q6CDJ7 (YALI0B23408p), and Q6CHE0 (glycine cleavage system P protein) (Figure 2b) exemplify expression changes with similar patterns in both strains, and thus such profiles were not considered in the current study. By this approach, 59 proteins changed abundance upon maximized lipid accumulation (*p*-value < 0.01, |log2 FC| > 1), 35 of which were upregulated (Figure 2c and Table S1, Supplementary File S4). Table 2 shows the top 10 proteins with largest differential expression (ranked by Euclidean distance) at maximized lipid yield.

The annotations of the most significantly downregulated proteins were insufficient to construe their biological significance, even though they might be important for further characterization. Among the remaining upregulated proteins at maximized lipid yield were several known dehydrogenases, including formate dehydrogenase (FDH) and alcohol dehydrogenase (ADH). Both enzymes are well known for their role in cofactor recovery, t mainly to promote NAD(P)H-dependent enzymes that are in high demand for lipid biosynthesis [2,11]. Moreover, conversion of dihydroxyacetone phosphate (DHAP) to glyceraldehyde-3-phosphate (G3P) by upregulated triose phosphate isomerase could possibly enhance lipid accumulation. This could promote the activity of glyceraldehyde-3-phosphate dehydrogenase (GAPDH), the NADH product of which can be transformed into NADPH in the cytoplasm through the pyruvate–oxaloacetate–malate cycle [12].

Lately, a dual role for the influence of reactive oxygen species (ROS) on lipid accumulation oleaginous microorganisms has been suggested. While the mechanism is largely unknown, balanced levels of ROS are reported to be favored in lipid accumulation [13]. On the other hand, high levels of ROS enhance lipid peroxidation. In general, generation of ROS is inevitable as byproducts of aerobic metabolism in high lipid production conditions. Moreover, lipid overproducers are highly exposed to oxidative stress, as reducing power is mostly driven to fatty acid biosynthesis, rather than being available for protection against oxidative stress [14]. Superoxide dismutase (SOD) acts as the first line of defense against ROS, and the upregulation of this enzyme observed here shows the importance of ROS stress response in lipid accumulation in this yeast.

**Figure 3:**
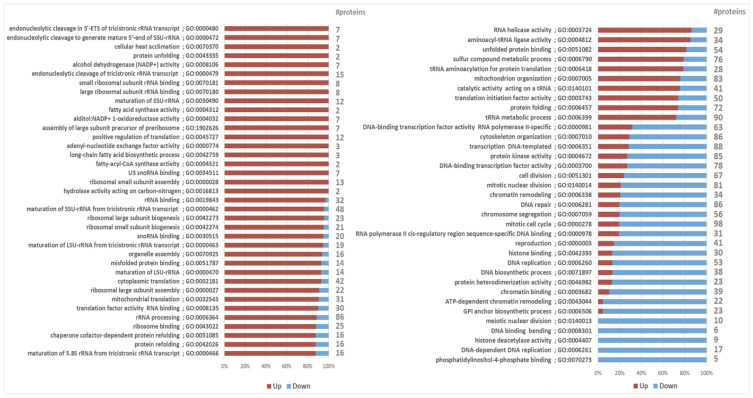
Gene set analysis of protein expression at maximized lipid yield. Protein sets are defined by GO terms. For each GO term showing significant enrichment (in this figure: adjusted *p*-value < 0.02), the direction of the relative changes in protein levels are shown, together with the total number of proteins within each GO term. Note that GO term annotations are redundant and the same genes are likely members of multiple GO terms.

To gain a pervasive view on the differential proteome composition and to put single protein alterations into a larger biological context, gene set analysis (GSA) was performed on all normalized protein abundances, while again focusing on the expression changes at maximized lipid yield. To accomplish this, all proteins were defined by Gene Ontology (GO) term annotations obtained from various databases. Using the created *Y. lipolytica* GO dataset consisting of 5509 GO terms (Table S2, Supplementary File S4), GSA showed 59 and 37 GO terms to be significantly (adj-*p*-value < 0.05) up- and downregulated at maximized lipid yield, respectively (Figure 3, Table S3, Supplementary File S4).

Overall, the maximum lipid yield seemed to coincide with *Y. lipolytica* tending to maximize its cofactor regeneration capacity, as well as organizing its stress responses. Mostly, the upregulated GO terms were involved in direct lipogenesis, oxidoreduction, protein folding, and ribosome biogenesis, whereas downregulated GO terms were mainly related to growth and cell division (Figure 3).

Guided by the overall GSA results, we identified a selection of novel proteomic responses that could help to further unravel the regulation of lipid accumulation, and we investigated these protein sets in more detail by examining the expression profiles of their individual proteins.

#### 2.2.1. Alternative NAPDH Suppliers for Lipid Accumulation

According to the GSA results, protein sets annotated with the GO terms alditol:NADP^+^ 1-oxidoreductase activity (GO:0004032), alcohol dehydrogenase (NADP^+^) activity (GO:0008106), and D-threo-aldose-1-dehydrogenase activity (GO:0047834) were significantly upregulated. While many of these proteins have previously been characterized to play a role in lipid accumulation, we were able to implicate several new proteins in providing NADPH for lipid production (Table S4, Supplementary File S4).

Among the most upregulated proteins were two proteins annotated as formate dehydrogenases (FDH) (Q6CDN8 and Q6C5X6), where both were present with high abundance. Q6C5X6 was the protein with the lowest *p*-value (2.3 × 10^−9^) among all analyzed proteins, firmly supporting the biologically significant role of FDH upregulation. Indeed, these two FDHs were found to significantly correlate with fatty alcohol production in a *Y. lipolytica* transcriptomic study [15]. Other *Y. lipolytica* genes encoding for FDHs have been reported to be upregulated in lipid-accumulating *Y. lipolytica* at low pH [16]. However, the biological significance of the upregulation remains unclear.

FDH is an NAD(P)^+^-dependent enzyme that catalyzes the reversible reaction between formate and carbon dioxide. Accordingly, the importance of NADPH in lipid biosynthesis is well known. Being highly reduced molecular species, overproduction of neutral lipids requires large quantities of NADPH, which is believed to be provided mainly by the pentose phosphate pathway (PPP) and malic enzyme [17]. The mannitol cycle has also been suggested to play a role in providing reducing power for neutral lipid overproduction [18]. Furthermore, enzyme engineering of FDHs with exclusive preference for NAD^+^, to broaden its preference to encompass NADPH regeneration, has been shown to improve lipid production [19]. From these results, it transpires that FDH might indeed play a significant role in lipid accumulation by providing reducing power, and here we identified two new candidate FDHs involved in this process.

Besides FDH, an NADP^+^-dependent isocitrate dehydrogenase (Q6C2Y4) and two NADP^+^-dependent glyoxylate reductases (Q6C7U5, Q6C284) were shown to be upregulated, suggesting complementary pathways to provide sufficient reducing power for lipid accumulation in *Y. lipolytica*.

#### 2.2.2. Boosting Precursor Supply

Well-known key lipogenic enzymes, such as fatty acid synthase (FAS), ATP citrate-lyase (ACL), and acetyl-CoA carboxylase (ACC), were significantly upregulated at the maximum lipid yield. In addition, several enzymes related to acetyl-CoA and other lipid precursors were differentially expressed (Table S5, Supplementary File S4). For instance, all subunits of the pyruvate dehydrogenase (PDH) complex were upregulated (GO:0006086, *p*-value 9 × 10^−4^ for all proteins). PDH catalyzes the conversion of pyruvate to acetyl-CoA, the lipid biosynthesis precursor, with lower energy costs in comparison to other strategies, such as via ACLs [20]. It is important to note that previous studies have shown that the main genes governing fatty acid synthesis are not directly controlled at the transcriptomic level [21,22].

Acetyl-CoA synthetase (Q6C2Q5) was also among the most significant upregulated proteins (*p*-value 7.92 × 10^−6^), which produces acetyl-CoA from acetate in the cytosol. Dihydrolipoyllysine-residue succinyltransferase (Q6C5L8) is upregulated (*p*-value 8.6 × 10^−2^) at maximized lipid yield. This enzyme subunit is part of oxoglutarate dehydrogenase but also involved in the degradation of L-lysine to acetyl-CoA via saccharopine. In particular, the latter function may boost the acetyl-CoA pool required for lipid biosynthesis. Meanwhile, significant downregulation (*p*-value 2.8 × 10^−6^) of acetyl-CoA hydrolase (Q6C0C0) minimized the degradation of this lipid precursor. Downregulation of other proteins also likely contributed to increasing the lipid biosynthesis precursor pool. For instance, biotin synthase (Q6C903), catalyzing the last step of de novo synthesis of biotin, was significantly downregulated (*p*-value 5 × 10^−3^). While downregulation of biotin synthesis seems counterintuitive as it is essential for ACC activity, it should be noted that the growth medium used in our study was supplemented with biotin, while the de novo biosynthesis of biotin shares precursors with fatty acid biosynthesis [23].

Therefore, enhancing precursor supply and redox cofactor regeneration in parallel transpired to be the most important metabolic strategies by which oleaginous *Y. lipolytica* was able to redirect its carbon flux and maximize its lipid yield.

#### 2.2.3. The Link between Unfolded Protein Response (UPR) and Lipid Accumulation

Among the significantly upregulated protein sets were eight that were assigned with GO terms related to protein folding. When inspecting the constituent proteins, these sets mostly contained proteins with roles as endoplasmic reticulum (ER)-located molecular chaperones, protein-folding enzymes, and those involved in cellular responses to unfolded proteins (Table S6, Supplementary File S4).

Accumulation of unfolded protein in the ER lumen causes ER stress, which in turn stimulates the unfolded protein response (UPR) via a conserved mechanism among eukaryotes. However, lipid overproduction is unlikely to accompany ER accumulation of unfolded proteins, and accordingly, comparison of the total cellular protein content denoted that maximum lipid yield indeed coincided with lower protein contents (Figure S2b, Supplementary File S3). Instead, also oxidative stress and accumulation of reactive oxygen species (ROS) are known to trigger ER stress [24] as a result of lipid oxidation and peroxidation in lipid overproducers [13,25].

Coinciding with upregulation of ER stress-related proteins was significant downregulation of proteins with ER–plasma membrane (PM) tethering activity (Table S2), including Q6CA74 (*p*-value 9.81 × 10^−5^), Q6C3Y8 (*p*-value 3 × 10^−4^), and Q6CA78 (*p*-value 3.6 × 10^−3^). Recent studies show that ER-PM junctions might have roles distinct from protein translocation into the ER [26,27,28]. More importantly, yeast cells lacking proteins for the ER-PM junction exhibited continuous UPR signaling, suggesting that functional ERs depend on ER-PM contact [29,30]. Therefore, upon loss of ER-PM signaling, cells may become dependent on other cell signaling and stress response systems, such as the UPR, to maintain ER homeostasis. While full elucidation of the role of ER-PM junction loss in lipid-accumulating *Y. lipolytica* requires further studies, our results demonstrated a co-occurrence of ER stress and UPR that could potentially be attributed to lipid accumulation.

Indeed, in *Saccharomyces cerevisiae*, ER stress has been shown to activate lipid droplet formation [31]. In a number of recent studies performed mainly on human cells, lipid accumulation was also shown to lead to ER stress, which along with UPR played essential roles in maintaining metabolic and lipid homeostasis and regulation [32,33,34,35]. Regardless, the current knowledge on these mechanisms is far from complete. Moreover, none of the *Y. lipolytica* orthologues of human UPR regulators involved in lipid metabolism [34] were shown to be differentially expressed in our data, suggesting that the link between UPR and lipid accumulation might be regulated at the phosphoproteomic level.

Among other members of the protein folding protein sets, several annotated to chaperone-mediated autophagy (CMA) were shown to be upregulated (Q6C0E9, *p*-value 1.2 × 10^−2^; Q6C3G5, *p*-value 1.2 × 10^−5^; Q6C864, *p*-value 6.3 × 10^−5^; Q6CCN4, *p*-value 1.8 × 10^−6^). CMA is the most selective form of autophagy that maintains cellular proteostasis in response to diverse stress conditions [36]. Previously, major forms of autophagy (macro- and microautophagy) have been reported to be upregulated in *Y. lipolytica* grown in nitrogen limitation in comparison to carbon limitation [21]. In the present study, where all cultivations were experiencing nitrogen limitation, the autophagy protein set was not differentially expressed. This suggests that CMA specifically correlates with maximum lipid yield, on top of general regulation of autophagy that would be observed during nitrogen limitation. Moreover, ER stress is known to activate CMA via a process named ERICA for ER stress-induced chaperone-mediated autophagy, a large part of which is still elusive [36]. It is also associated with the induction of UPR, and therefore could be linked with lipid accumulation via the ER stress–UPR axis.

#### 2.2.4. Sulfur Amino Acid (SAA) Metabolism May Affect Lipid Accumulation

Sulfur compound metabolic process (GO:0006790) was among the significantly upregulated protein sets and contained proteins with SAA metabolism activity (Table S7, Supplementary File S4). Most members of this protein set whose expression was significantly changed at maximum lipid yield were involved in methionine (Met) and homocysteine (Hcy) biosynthesis. The link between methionine and lipid biosynthesis in yeasts has only been reported by a handful of studies. Other than a study where methionine addition increased palmitoleic acid production in *S. cerevisiae* [37], He et al. reported the upregulation of one gene (MET17) in methionine biosynthesis by studying the transcriptional profile of an oleaginous strain of *S. cerevisiae* in comparison to a non-oleaginous strain [38]. Another line of evidence suggests that Met metabolism can enhance oxidative stress resistance in *S. cerevisiae* by upregulating the NADPH-producing PPP [39].

Met and Hcy metabolism has also been linked to lipid accumulation in human-related studies, where elevated intracellular levels of Hcy triggered ER stress [40], which is known to promote lipid accumulation, as previously described.

While downregulation of amino acid biosynthesis upon nitrogen limitation and lipid accumulation has been reported in *Y. lipolytica* [21], current data suggest that—besides the redirection of carbon flux from amino acid biosynthesis to lipid overproduction—an active regulation to specific amino acids (such as methionine) might positively affect the lipid yield.

#### 2.2.5. Other Differentially Expressed Proteins and Protein Sets

Various other protein sets were found to be differentially regulated at maximum lipid yield (Figure 3). Of these, proteins involved in ribosome biogenesis and function were shown to be upregulated. Although more likely associated with growth rather than lipogenesis, our linear modeling approach suggested that there might be some interconnections with lipid biosynthesis. Noteworthy is that the cellular protein content was reduced at the maximum lipid yield (Figure S2, Supplementary File S2), such that a downregulation of ribosomal proteins would have been anticipated. The upregulation of protein sets with rRNA and translation-related proteins can therefore denote translational regulation of lipid metabolism. Moreover, although ribosomal proteins are mainly considered to be involved in protein production, some of them are known to have pleiotropic functions that mediate a wide range of cell homeostasis-regulatory roles. Accordingly, there are reports that several ribosomal proteins activate UPR [41,42,43,44], whose role in lipid biosynthesis regulation is described above.

Arginase (Q6C6P6) is a member of the upregulated protein set of nonpeptide amidine hydrolases (GO:0016813). When inspecting its expression, a complex pattern appeared (Figure 2b): while downregulated at lower dilution rates, this downregulation was far less profound in the lipid-accumulating strain, so that it was identified as relatively upregulated at maximum lipid yield. Moreover, the relative abundance of this protein was almost triple in the control strain. Through its role in the urea cycle, arginase releases ammonium, and with nitrogen limitation as inducer of lipid accumulation, a lower abundance of arginase could generally be beneficial at maximized lipid yield.

Among the downregulated protein sets was protein kinase activity (GO:0004672), containing several hypothetical regulators. HOG1 (Q6C4M9, *p*-value 1 × 10^−3^) is a mitogen-activated protein kinase (MAPK) primarily characterized to be involved in its response to osmotic stress. Recently, a role of HOG1 in controlling lipid homeostasis in *Candida albicans* was shown as *hog1* mutants accumulated lipid droplets [45]. Furthermore, in *S. cerevisiae*, HOG1 was suggested to control fatty acid mobilization and beta-oxidation upon salt addition or glucose deprivation [46]. Although no osmotic stress was exerted in our current study, the meaningful downregulation of HOG1 at maximum lipid yield makes it an interesting target for further analysis. Besides being a well-known osmoregulation factor, more diverse roles have been proposed for HOG1, especially in different stress responses [45], somewhat increasing the possibility of its involvement in regulating lipid biosynthesis.

Although autophagy protein sets were not differentially expressed in the current study, a few autophagy-related proteins were shown to be significantly downregulated. This included Q6C375, a hypothetical protein with sequence similarity to *S. cerevisiae* MDM1, which is proposed to maintain yeast cellular homeostasis by promoting the incorporation of lipids in lipid droplets for efficient delivery to the vacuole for lipophagy. This is in line with maximum lipid yield in *Y. lipolytica* coinciding with downregulation of this protein (*p*-value 2 × 10^−3^). Also, in other organisms, e.g., human hepatic cells, it was shown that the knockdown of some autophagy-related genes can increase neutral lipid levels [47].

### 2.3. Strain Engineering

Guided by the results from GSA and analysis of its protein sets, six proteins were selected from diverse protein sets with high significance scores, solid biological relevance, and novelty in terms of their role in lipid accumulation. Their role in lipid accumulation was investigated by genetic engineering of either knockout or overexpression (Table 3).

**Figure 4:**
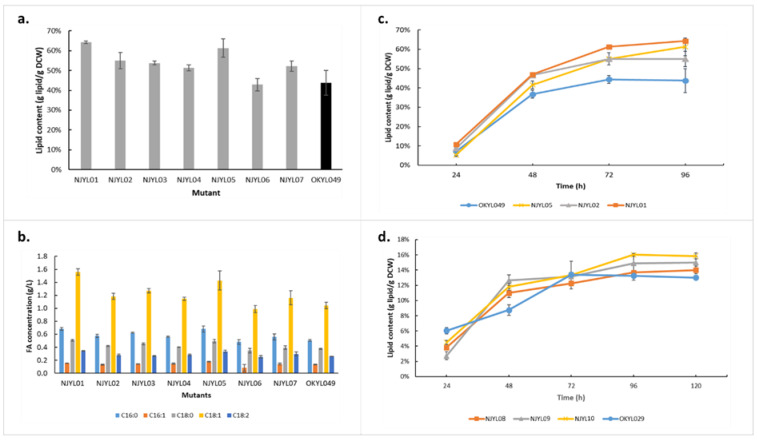
Lipid phenotype of *Y. lipolytica* after genetic engineering of selected targets. Lipid content (**a**) and fatty-acid composition (**b**) of 7 mutants cultivated for 96 h in nitrogen-limited shaken flasks in comparison to *Y. lipolytica* OKYL049. (**c**) Time-course lipid content analysis of the three most promising mutants with OKYL049 as control. (**d**) Time-course lipid content analysis of mutants with the same genetic targets as in (**c**), but now implemented in the OKYL029 background. Data are presented as the average of three independent experiments, Error bars represent means ± standard deviation.

Using the lipid-accumulating OKYL049 as parental strain, NJYL01 to NJYL07 strains were constructed (See Section 3) and their fatty acid contents evaluated from shake-flask cultivations after 96 h (Figure 4a). It is noteworthy that this approach focuses all mutants, except for FDH overproducers producing higher amounts of lipid (*p*-value < 0.05). Although FDH was among the most significantly upregulated proteins at maximum lipid yield, overexpression of this gene did not increase lipid production. Meanwhile, a somewhat higher lipid content was observed when a stronger promoter sequence was used (cf. NJYL06 and NJYL07, Figure 4a), suggesting that FDH overexpression was insufficient to invoke a significant increase in lipid content. The total fatty acid composition in all mutants followed the same pattern as its parental strain (Figure 4b). NJYL01, NJYL02, and NJYL05, representing knockdowns of biotin synthase, arginase, and HOG1, respectively, showed the biggest increases in lipid production among all mutants. The three mutants had roughly 23%, 14%, and 20% higher lipid contents than their parental strain after 96 h (Figure 4a). Additional time-course measurements of these promising strains showed consistently higher lipid content (Figure 4c).

As the lipid-accumulating parental strain OKYL049 was already engineered to overproduce lipids, it might have already approached its limits after rewiring of native lipogenesis regulation. Therefore, we also implemented the same three most promising genetic mutations in the OKYL029 strain (parental to OKYL049) that did not already contain mutations affecting lipid metabolism, yielding strains NJYL08–10. All three mutants, i.e., knockouts of biotin synthase, arginase, and HOG1, resulted in faster initiation of lipid accumulation, as indicated by a sharper slope until 48 h (Figure 4d). However, these genetic mutations were far from sufficient to reach the lipid content observed in the OKYL049 strain, implicating that these genetic strategies are complementary to direct rewiring of lipid biosynthesis. While we only selected a handful of genes for validation by genetic manipulation, the observed results (Figure 4) strongly support most of the conclusions that were drawn from the proteomic data.

Conclusively, our strategy of elucidating how growth rate affects lipid accumulation by using a factorial experimental design with a non-lipid-accumulating control strain and a generalized linear model using proteomic data has proven successful. This is also important, since in transcriptional profiling, the commonly utilized strategy for identifying genes associated with certain biological processes, such as lipid biosynthesis, the correlation between transcription and translation is known to be generally low. The mechanisms identified through this study were shown to be vastly variable in terms of biological context, demonstrating a systematic orchestration of cellular function for maximized lipid yield. In particular, other than prereported proteins, we were also able to elucidate novel proteins with roles in precursor supply and redox cofactor regeneration. More importantly, nonintuitive biological functions, such as the contribution of ER stress and UPR in oleaginousness of *Y. lipolytica* was shown. To the best of our knowledge, such a link has not previously been reported in oleaginous yeasts. While proposed in human-based studies, the mechanism of the contribution of UPR and ER stress in lipid metabolism is still elusive and is beginning to be appreciated. Understanding such links may provide guidance for the development of stress-based strategies to enhance microbial lipid production. Moreover, while most of the identified proteins and protein sets could be directly linked to lipid metabolism, several others were more indirect, such as the roles of arginase, HOG1, chaperone-mediated autophagy proteins, and ER-PM tether proteins. Characterization of their mechanisms requires further investigations. Nonetheless, this work provides rich data on the biological events leading to growth rate-dependent lipid accumulation in *Y. lipolytica*. This proteomic information should be valuable for studies related to rational engineering of *Y. lipolytica* and other oleaginous yeasts.

## 3. Materials and Methods

### 3.1. Yeast Strains, Cultivation, and Sample Collection

Table 4 shows all strains used in this study, which are derived from the *Yarrowia lipolytica* strain ST6512 [48]. ST6512 is in turn derived from the W29 background strain (Y-63746 from the ARS Culture Collection, Peoria, IL, USA; a.k.a. ATCC20460/CBS7504) which has been engineered to harbor a KU70::Cas9-DsdA to allow fast marker-free genomic engineering using the EasyCloneYALI toolbox [49].

*Y. lipolytica* OKYL049 and *Y. lipolytica* JFYL007 (Q4) (hereafter referred to as OKYL049 and Q4, respectively) were used as the main strains for the comparative analysis. Our Q4 strain is analogous to the earlier reported strain [8,9,10], but implemented in the same OKYL029 parental strain as OKYL049. While Q4 strain lacks diacylglycerol acyltransferases genes and unable to accumulate lipids, OKYL049 is a lipid overproducer due to the introduction of DGA1 over expression by deletion of ARE1 to increase TAG accumulation and abolish sterol ester formation [7].

All chemostat experiments were performed under nitrogen-limited conditions on minimal mineral (MM) medium containing per liter: 25 g of glucose, 0.47 g (NH_4_)_2_SO_4_, 3 g KH2PO4, 0.5 g MgSO_4_·7H_2_O, 0.2 mL antifoam 204 (Sigma, Gothenburg, Sweden), as well as 1 mL vitamin solution and 1 mL trace metal solution prepared as mentioned elsewhere [51]. The chemostat fermentations were performed in 1.2 L DASGIP Bioreactors (Dasgip, Jülich, Germany), equipped with off-gas analysis, pH, temperature, and dissolved oxygen sensors with the following conditions: working volume 0.5 L, 28 °C, airflow of 1 vvm with 21% O_2_, pH 5 controlled with addition of 2 M KOH and dissolved oxygen of 30% via feedback control of agitation from 400 rpm to a maximum of 1200 rpm. The concentration of O_2_ and CO_2_ in the off-gas were measured using the DASGIP GA4 exhaust analyzer after being cooled by a condenser operated at 4 °C. To select optimum growth rates for comparative analysis, different dilution rates ranging from 0.02 to 0.16 h^−1^ were used to run chemostat experiments. Comparative analysis was then performed based on data from chemostat fermentations at 0.06 and 0.1 h^−1^.

To ensure cells were growing at a steady state, chemostats were run for at least five residence times defined as stable CO_2_ and O_2_ outflow before sampling at each dilution rate. After discarding the dead volume of the sampling port, samples were taken for cell dry weight (CDW), glucose and organic acid quantification, lipid production analysis via fatty acid methyl esters (FAME) measurement, and proteomic analysis. CDW measurements were started by vacuum filtration of cell broth on preweighed 0.45 μm polyethersulfone (PES) membranes (Sartorius) which were heated via microwaving at 125 to 325 W for 15 min and placed in a desiccator for at least 2 days before measuring the weight increase, which was later determined and normalized into gDW/L. Sampling for analysis of metabolites in extracellular medium was performed by filtration with 0.45 μm filters and storage of cell-free culture liquid at −20 °C. Samples for lipid and FAME measurements were taken in the form of 2 mL cell broth aliquots, which were centrifuged, the supernatant was discarded, and the cells were washed twice with 1 mL water. They were lyophilized in form of fluffy cell pellets and stored in −20 °C until analysis. For proteomic analysis, samples were collected in tubes chilled on ice and further centrifuged for 4 min at 3000× *g* at 4 °C; cell pellets were washed once with 20 mL of chilled dH_2_O, flash frozen in liquid nitrogen, and stored at −80 °C until analysis. Samples of each experiment were taken from three independent chemostats.

Shake-flask cultivation was used to allow the simultaneous evaluation of cell growth and lipid accumulation of strains in this study with those observed for control strains (OKYL049 or OKYL029). The experiments were performed in triplicate and the CDW as well as FAME samples were taken every 24 h and prepared for measurement as mentioned before. Cultivations lasted for maximum of 120 h when the glucose was exhausted in the fermentation media. Accordingly, the strains were cultured in 25 mL MM (slightly modified: phosphate buffer enhanced to 0.2 M) and with C/N ratio 100 to exert nitrogen limitation incubated at 30 °C at 200 rpm.

### 3.2. Strain Construction

All *Y. lipolytica* strains constructed in this study are derived from ST6512, which are the wild-type W29 (Y-63746 from the ARS Culture Collection, Peoria, IL, USA; a.k.a. ATCC20460/CBS7504)-derived strain harboring Cas9 in KU70 locus to allow fast marker-free genomic engineering using the EasyCloneYALI toolbox. OKYL029 and OKYL049 were used as the parental strains. The complete list of plasmids and primers used in this work is available in the Supplementary File S1. The strains are available upon request.

Unless otherwise stated, for preculture, strain construction, propagation, and cryostocking, yeast strains were grown at 30 °C and 240 rpm (Thermo Fisher Scientific, Gothenburg, Sweden) in YPD medium (10 g/L yeast extract, 20 g/L peptone, 20 g/L D-glucose). Media for plates contained 20 g/L agar.

Plasmids required for genome engineering were constructed using the set of vectors from EasyCloneYALI as backbones [49]. Plasmid assembly and cloning was performed according to EasyCloneYALI instructions [49]. DNA fragments were amplified by PCR using Phusion U polymerase (Thermo Fisher Scientific), which were then were purified from agarose gels using the NucleoSpin gel and PCR cleanup kit (Macherey-Nagel, Gothenburg, Sweden). The DNA fragments were assembled into EasyCloneYALI vectors using USER cloning.

For gene deletions of YALI0D05291p, YALI0D15400g, YALI0E07535g and YALI0F02035p, repair templates were obtained from equal amounts of two single-stranded oligonucleotides (around 120 bp; 100 pmol/μL), which were incubated for 5 min at 98 °C and allowed to cool down to room temperature. For gene deletion of YALI0E25135g, the DNA fragments consisting of around 450 bp up- and downstream of the target gene were obtained by fusion PCR and used as the repair template. The guide-RNA (gRNA) vectors for the 5 genes above were constructed using USER cloning methods with corresponding primers. For overexpression of YALI0E14256, two strong promoters, pTef and pTefintro were used. The pTef-YALI0E14256 and pTef-YALI0E14256 fragments were integrated into digested backbone pCfB6684, and the obtained plasmids were digested by NotI and inserted into D1 locus with the gRNA plasmid pCfB6631.

All constructed plasmids were verified by Sanger sequencing (Eurofins Scientific SE, Gothenburg, Sweden). Yeast transformations were performed using a lithium acetate-based protocol as previously described [49]. Transformants were selected using natMX or hphMX resistance markers. YPD plates for natMX selection contained 250 mg/L of nourseothricin (Jena Bioscience, Malmo, Sweden).

### 3.3. Exometabolic Analysis

To quantify glucose concentrations in medium and other extracellular metabolites where needed, fermentation samples were prepared by taking 1 mL of culture, centrifuging for 5 min at 3000× *g*, and using the supernatant for high-performance liquid chromatography (HPLC) analysis. The HPLC system UltiMate 3000 (Dionex, Stockholm, Sweden) was utilized with an Aminex HPX-87H ion exclusion column (Bio-Rad, Solna, Sweden). 5 mM H_2_SO_4_ was used as eluent at a flow rate of 0.6 mL/min. Glucose and xylose were quantified using a refractive index detector (Shodex, Munich, Germany).

### 3.4. Lipid Extraction and Quantification

To measure cellular lipid content, fatty acids were extracted and derivatized to fatty acid methyl esters (FAMEs) and subsequently analyzed by GC-MS using a previously published method [52]. Briefly, 100 μg of C17:0 TAG internal standard was added to the lyophilized cell pellets. Samples were vortex at 1200 rpm at room temperature for 1 h after addition of 500 μL of methanol solution containing 1M NaOH. The solution was then neutralized by adding 160 μL of 49% sulfuric acid. Finally, by addition of 500 μL hexane FAMEs were extracted. After centrifugation at 10,000× *g* for 1 min, phases were separated and 100 μL of the upper hexane phase was mixed with 900 μL hexane, 1 μL of which was autoinjected for analysis on GC-MS (Thermo Scientific Trace 1310 coupled to a Thermo Scientific ISQ LT) with a ZBFAME column (Phenomenex, length: 20 m; Inner Diameter: 0.18 mm; Film Thickness: 0.15 μm).

C16:0, C16:1, C18:0, C18:1 and C18:2 fatty acids (FAs) were considered for the calculation of lipid content per cell dry weight (g fatty acid/g dry biomass) as well as the contribution of each FA to the total FA content. To this end, dilution series of the FAME mixture standard, GLC-403 (Nu-Chek Prep, Elysian, MN, USA) was used as external standards analyzed along with samples under similar conditions.

### 3.5. Proteomic Analysis

#### 3.5.1. Protein Extraction

Cell pellets were homogenized using a FastPrep-24 instrument (MP Biomedicals) with lysing matrix D for five repeated cycles (speed 6.5 m/s, 40 s/cycle) in 200 µL of the buffer containing 3% sodium dodecyl sulfate and 50 mM triethylammonium bicarbonate (TEAB). Samples were centrifuged at 16,000× *g* for 10 min and the supernatants were transferred to clean tubes. The lysis tubes were washed with 100 µL of the lysis buffer, centrifuged at 16,000× *g* for 10 min, the supernatants were combined with the corresponding lysates from the previous step. Protein concentration in the combined lysates was determined using a Pierce BCA protein assay kit (Thermo Scientific) and the Benchmark Plus microplate reader (Bio-Rad) with bovine serum albumin (BSA) solutions as standards. A representative reference sample was prepared, containing equal amounts from the 12 individual samples

#### 3.5.2. Tryptic Digestion and Tandem Mass Tag (TMT) Labeling

Aliquots containing 30 μg of each sample including the reference were reduced with 100 mM DL-dithiothreitol (DTT) at 56 °C for 30 min. The reduced samples were processed using the modified filter-aided sample preparation (FASP) method [53]. In short, reduced samples were transferred to 30 kDa MWCO Pall Nanosep centrifugation filters (Pall Corporation, Lund, Sweden) and washed three times with 8 M urea. Additional washes with digestion buffer (1% sodium deoxycholate in 50 mM TEAB) were performed before and after alkylation with 10 mM methyl methanethiosulfonate for 20 min at room temperature. Protein digestions were performed using trypsin (Pierce MS grade) in digestion buffer, first with 0.3 µg trypsin at 37 °C for 4 hours followed by new addition of 0.3 µg trypsin and incubation at 37 °C overnight. Produced tryptic peptides were collected by centrifugation and labeled using tandem mass Tag (TMTpro 16plex) reagents (Thermo Fisher Scientific) according to the manufacturer’s instructions. The labeled samples were combined into one pooled sample, concentrated using vacuum centrifugation, and SDC was removed by acidification with 10% TFA and subsequent centrifugation. The labeled pooled sample was treated with Pierce peptide desalting spin columns (Thermo Fisher Scientific) according to the manufacturer’s instructions.

The combined TMT-labeled sample was fractionated into 40 primary fractions by basic reverse-phase chromatography (bRP-LC) using a Dionex Ultimate 3000 UPLC system (Thermo Fisher Scientific). Peptide separations were performed using a reversed-phase XBridge BEH C18 column (3.5 μm, 3.0 × 150 mm, Waters Corporation, Solna, Sweden) and a linear gradient from 3% to 40% solvent B over 18 min followed by an increase to 100% B over 5 min and 100% B for 5 min at a flow of 400 µL/min. Solvent A was 10 mM ammonium formate buffer at pH 10.00 and solvent B was 90% acetonitrile, 10% 10 mM ammonium formate at pH 10.00. The first 18 primary fractions were concatenated into 9 fractions (1 + 10, 2 + 11, …) and following fractions were left separately. All were evaporated and reconstituted in 20 μL of 3% acetonitrile, 0.2% formic acid for nLC-MS/MS analysis.

#### 3.5.3. nLC-MS/MS

The fractions were analyzed on an Orbitrap Fusion Tribrid mass spectrometer interfaced with Easy-nLC1200 liquid chromatography system (Thermo Fisher Scientific). Peptides were trapped on an Acclaim Pepmap 100 C18 trap column (100 μm × 2 cm, particle size 5 μm, Thermo Fisher Scientific) and separated on an in-house packed analytical column (75 μm × 40 cm, particle size 3 μm, Reprosil-Pur C18, Dr Maisch, Ammerbuch, Germany) using a linear gradient from 5% to 12% B over 5 min, 12% to 35% B over 70 min followed by an increase to 100% B for 5 min, and 100% B for 10 min at a flow of 300 nL/min. Solvent A was 0.2% formic acid and solvent B was 80% acetonitrile, 0.2% formic acid. MS scans were performed at 120,000 resolution, *m*/*z* range 375–1500. MS/MS analysis was performed in a data-dependent manner, with top speed cycle of 3 s for the most intense doubly or multiply charged precursor ions. Precursor ions were isolated in the quadrupole with a 0.7 *m*/*z* isolation window, with dynamic exclusion set to 10 ppm and duration of 45 s. Isolated precursor ions were subjected to collision-induced dissociation (CID) at 30 collision energy with a maximum injection time of 50 ms. Produced MS2 fragment ions were detected in the ion trap followed by multinotch (simultaneous) isolation of the top 10 most abundant fragment ions for further fragmentation (MS3) by higher-energy collision dissociation (HCD) at 55% and detection in the Orbitrap at 50,000 resolution, *m*/*z* range 100–500.

#### 3.5.4. Proteomic Data Analysis 

The data files of the set were merged for identification and relative quantification using Proteome Discoverer version 2.4 (Thermo Fisher Scientific). Identification was performed using Mascot version 2.5.1 (Matrix Science, London, UK) as a search engine by matching against the *Yarrowia lipolytica* database of SwissProt (November 2020). The precursor mass tolerance was set to 5 ppm and fragment mass tolerance to 0.6 Da. Tryptic peptides were accepted with 0 missed cleavage; methionine oxidation was set as a variable modification, cysteine methylthiolation, TMTpro on lysine and peptide N-termini were set as fixed modifications. Percolator was used for PSM validation with the strict FDR threshold of 1%.

TMT reporter ions were identified in the MS3 HCD spectra with 3 mmu mass tolerance, and the TMT reporter intensity values for each sample were normalized within Proteome Discoverer 2.4 on the total peptide amount. Only the unique identified peptides were considered for the relative quantification. A reference sample made from a mix of all the samples were used as denominator and for calculation of the ratios.

Differentially expressed proteins were analyzed using DEqMS which is developed on top of Limma R package [54] while modifying Limma’s variance prior estimation to consider the dependence of variance on the number of detected peptides/PSMs per protein [55]. Considering the number of PSMs, a more accurate data-dependent estimation of protein variance is allowed and at the same time the inclusion of single peptide identifications without increasing false discoveries will be possible.

Since there are two experimental dimensions being varied in this analysis (i.e., strain and dilution rate), a 2 × 2 factorial design was used. Accordingly, a model matrix with so called treatment contrast parameterization was created as follows:

Dilution rate = c(0,0,1,1,1,0,0,0,1,1)

Strain = c(1,1,1,1,1,0,0,0,0,0)

> design <- model.matrix (~Strain*Dilution rate)

where 0 represents the higher dilution rate and control train, while 1 represents the OKYL049 and lower dilution rate.

Among the comparisons the design matrix created, results from interaction term were used for further analysis. The interaction term will show which proteins are differentially expressed in OKYL049 compared to the control strain (Q4) in the lower dilution rate. The generated fold changes and *p*-values from the interaction model were further used as input data for gene/protein set analysis (GSA) using the “piano” package [56]. The mean of the fold changes as the set statistic and gene/protein permutation with false-discovery rate correction of the obtained *p*-values to estimate significance was used. The protein sets were generated based on GO term annotation dataset of the detected proteome of *Y. lipolytica* created using AmiGO [57], Blast2GO [58], Ensembl [59], InterPro [60] and UniProt [61]. The protein sets with more than two genes and fewer than 100 genes were selected for the analysis.

## Figures and Tables

**Table 1:** Results of the chemostat cultivation of *Y. lipolytica* OKYL049 at different dilution rates under nitrogen limitation.

Dilution Rate (h^−1^)	Residual Glucose (g/L)	q-Glucose (g/g/h)	Total Lipid (g/L)	Lipid Content (%)	Non-Lipid CDW (g/L)	CDW (g/L)
0.02	10.03 ± 0.63	0.1 ± 0.005	1.06 ± 0.05	30 ± 0.46	2.51 ± 0.11	3.57 ± 0.16
0.03	11.94 ± 0.98	0.1 ± 0.004	1.04 ± 0.08	28 ± 1.27	2.68 ± 0.05	3.72 ± 0.12
0.04	15.05 ± 0.47	0.12 ± 0.003	0.88 ± 0.06	25 ± 1.09	2.61 ± 0.05	3.49 ± 0.10
0.05	16.58 ± 0.17	0.14 ± 0.019	0.84 ± 0.05	26 ± 1.61	2.36 ± 0.18	3.20 ± 0.20
0.06	16.44 ± 0.16	0.16 ± 0.011	0.95 ± 0.05	27 ± 0.89	2.53 ± 0.03	3.48 ± 0.07
0.08	18.68 ± 0.40	0.19 ± 0.006	0.67 ± 0.08	23 ± 1.90	2.30 ± 0.20	2.97 ± 0.26
0.12	19.61 ± 0.10	0.27 ± 0.015	0.43 ± 0.05	20 ± 1.39	1.71 ± 0.10	2.14 ± 0.14
0.16	20.74 ± 0.17	0.33 ± 0.054	0.28 ± 0.03	17 ± 1.55	1.37 ± 0.03	1.66 ± 0.03

Values are means ± standard deviation of the mean.

**Table 2:** Top 10 proteins with the largest differential expression upon maximized lipid yield.

Protein Accession	Protein Annotation	GO (Molecular Function)	Change	log2 (FC)	−log10 (*p*-Value)
Q6C5X6	Formate dehydrogenase	formate dehydrogenase (NAD+) activity [GO:0008863]; oxidoreductase activity, acting on the CH-OH group of donors, NAD or NADP as acceptor [GO:0016616]	Up	2.48	8.64
Q6CAP1	-	carbohydrate:proton symporter activity [GO:0005351]	Up	3.66	7.88
F2Z678	Alcohol dehydrogenase 2	alcohol dehydrogenase (NAD+) activity [GO:0004022]; zinc ion binding [GO:0008270]	Up	1.81	8.42
Q6CI12	-	hydrolase activity, acting on carbon-nitrogen (but not peptide) bonds, in linear amidines [GO:0016813]; peptidase activity [GO:0008233]	Up	1.18	8.47
Q6C676	-	-	Up	1.69	7.71
Q6CBW3	-	-	Down	2.73	6.94
Q92389	Acid extracellular protease	aspartic-type endopeptidase activity [GO:0004190]	Up	2.37	7.04
Q6CBQ1	Superoxide dismutase	manganese ion binding [GO:0030145]; superoxide dismutase activity [GO:0004784]	Up	1.33	7.27
Q6C395	Triose phosphate isomerase	triose-phosphate isomerase activity [GO:0004807]	Up	2.12	6.99
Q6C8H3	-	hydrolase activity, hydrolyzing O-glycosyl compounds [GO:0004553]	Down	1.67	7.01

**Table 3:** Selected mutation targets for experimental analysis of lipid accumulation in *Y. lipolytica*.

Mutant (in OKYL049)	Mutant (in OKYL029)	Target Accession	Target Name	Biological Significance	FC Direction and *p*-Value	Mutation Strategy
NJYL01	NJYL08	Q6C903	Biotin synthase	Biotin shares biosynthetic precursors with lipids	Down, 5.4 × 10^−3^	Knockout
NJYL02	NJYL09	Q6C6P6	Arginase	Puts burden on cell energetically, produces ammonia to alleviate nitrogen limitation	Down, 5.4 × 10^−7^	Knockout
NJYL03		Q6CA74	PapD-like protein	ER-PM tether which maintains ER homeostasis	Down, 9.8 × 10^−5^	Knockout
NJYL04		Q6C375	Similar to MdM1	Role in lipophagy	Down, 2 × 10^−3^	Knockout
NJYL05	NJYL10	Q6C4M9	HOG1	A MAPK known to affect lipogenesis during osmotic stress	Down, 1.5 × 10^−3^	Knockout
NJYL06 and NJYL07		Q6C5X6	Formate dehydrogenase	NADPH is a key cofactor for lipid biogenesis	Up, 2.3 × 10^−9^	Overexpression, different promoters

**Table 4:** *Y. lipolytica* strains used in the current study.

*Y. lipolytica* Strain	Genotype	Reference
OKYL049	MATa, ∆ku70::Cas9::DsdA, lntE1::Tef1inp-DGA1PEX20t, Δare1Δmhy1	[50]
OKYL029	MATa, ∆ku70::Cas9::DsdA, Δmhy1	[7]
JFYL007 (Q4)	MATa, ∆ku70::cas9∆mhy1∆ARE1∆LRO1∆DGA1∆DGA2	Unpublished
NJYL01	MATa, ∆ku70::Cas9::DsdA, lntE1::Tef1inp-DGA1PEX20t, Δare1Δmhy1ΔYALI0D15400g	This study
NJYL02	MATa, ∆ku70::Cas9::DsdA, lntE1::Tef1inp-DGA1PEX20t, Δare1Δmhy1ΔYALI0E07535g	This study
NJYL03	MATa, ∆ku70::Cas9::DsdA, lntE1::Tef1inp-DGA1PEX20t, Δare1Δmhy1ΔYALI0D05291g	This study
NJYL04	MATa, ∆ku70::Cas9::DsdA, lntE1::Tef1inp-DGA1PEX20t, Δare1Δmhy1ΔYALI0F02035g	This study
NJYL05	MATa, ∆ku70::Cas9::DsdA, lntE1::Tef1inp-DGA1PEX20t, Δare1Δmhy1ΔYALI0E25135g	This study
NJYL06	MATa, ∆ku70::Cas9::DsdA, lntE1::Tef1inp-DGA1PEX20t, Δare1Δmhy1, lntD1::Tefp- YALI0E14256g-lip2t	This study
NJYL07	MATa, ∆ku70::Cas9::DsdA, lntE1::Tef1inp-DGA1PEX20t, Δare1Δmhy1, lntD1::Tefinp-YALI0E14256g-lip2t	This study
NJYL08	MATa, ∆ku70::Cas9::DsdA, Δmhy1 ΔYALI0D15400g	This study
NJYL09	MATa, ∆ku70::Cas9::DsdA, Δmhy1 ΔYALI0E07535g	This study
NJYL10	MATa, ∆ku70::Cas9::DsdA, Δmhy1 ΔYALI0E25135g	This study

## Data Availability

Data is contained within the article or Supplementary Material.

## Supplementary Materials

The supporting information can be downloaded at: https://www.mdpi.com/article/10.3390/ijms23158517/s1.
