# Supplementary material for: Validated Growth Rate-Dependent Regulation of Lipid Metabolism in Yarrowia lipolytica
Source: Int J Mol Sci. 2022 Jul 31;23(15):8517. doi: 10.3390/ijms23158517 (PMC9369070; doi:10.3390/ijms23158517)
Supplement: Supplementary file 1 [file ijms-23-08517-s001.zip › Supplementary File S2.pdf]

SUPPLEMENTARY FILE S1

1. Microscopic analysis of lipid droplets of OKYL049

A fraction of cells for each dilution rate were stained with 1 µL of BODIPY 493/503 (Thermo Fisher Scientific) solution (1 mg/mL in ethanol), which binds specifically to lipids and provides a green fluorescent signal. The stained yeast cultures were observed using a fluorescence microscope (Leica, DMI4000B) (Figure S1).

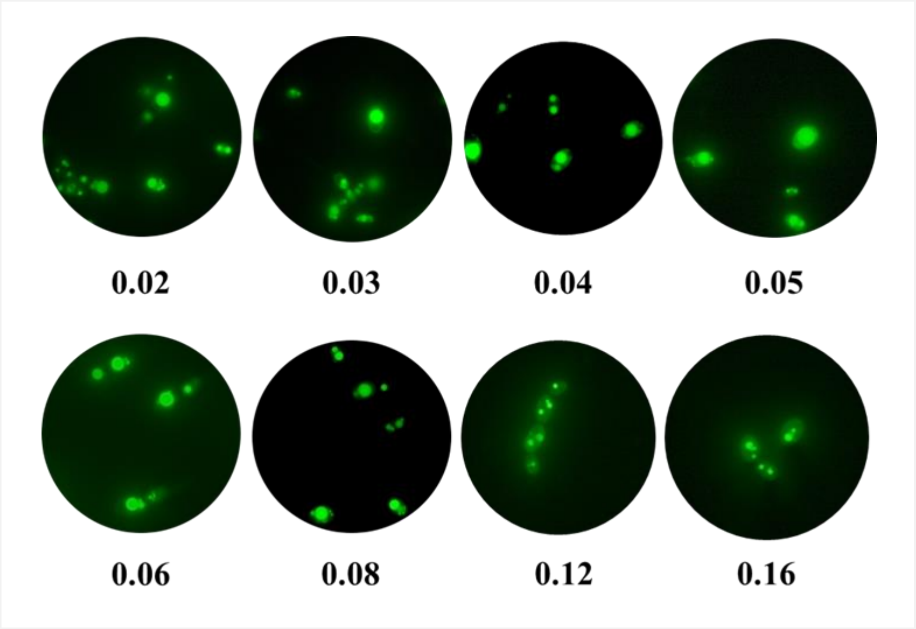

Figure S1. BODIPY staining of lipid bodies in OKYL049 grown at different growth rates (D 0.02 to D 0.16 h-1) which roughly shows bigger lipid droplets in D 0.06 and 0.05 in comparison to very low and very high dilution rates.

2. *Y. lipolytica* JFYL007 (Q4) as the control strain

Q4 seems to have less variation in terms of lipogenesis-related metrics in the two dilution rates as can be seen in Figure S2. Protein content analysis (method below) of the two strain also showed higher concentration in Q4 which is reasonable since in OKYL049 resources will be more directed to lipid biosynthesis limiting energy sources for higher protein levels. This can also be observed in lower dilution rate. However, changes in Q4 is not significant between the two dilution rate. Q4 makes a good candidate to be used as a control strain for comparative studies which is low to non-lipid producing without such drastic transient behavior.

Protein content measurement (BCA assay)

Protein extraction was initiated on a minimum of 2 mgDW biomass which were resuspended in 1ml of 2M NaOH followed by the addition of 1ml of 20% SDS and boiling for 10 minutes. After the addition of HAce (17M), the lysates were spun down at 12,000 g for 5 min, after which the supernatant was collected and diluted 5X in water. The concentration of protein in the supernatant was determined with a BCA Assay kit (Pierce™ BCA Protein Assay Kit, Thermofisher) using the use enhanced protocol via microplate procedure as instructed by the provider. For each chemostat bioreactor 3 independent samples were analysed.

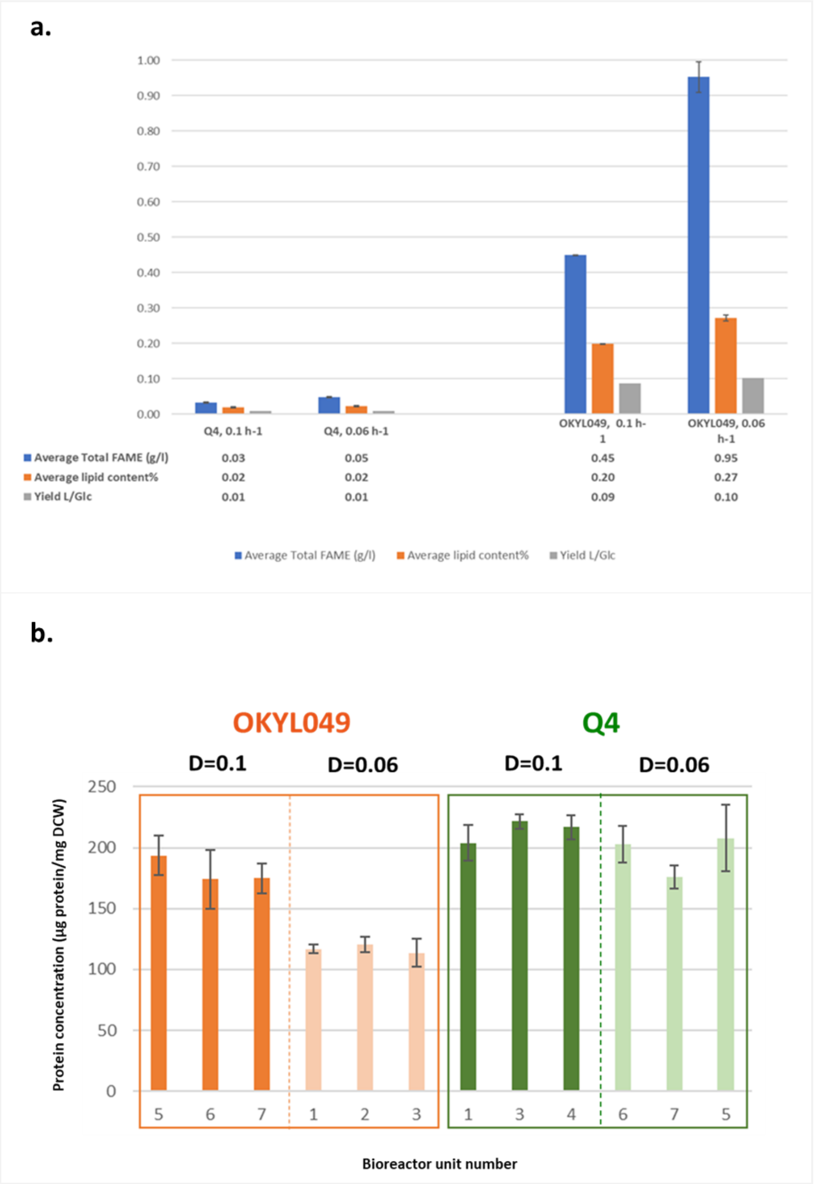

Figure S2. (a) comparison of lipid production metrices of OKYL049 and Q4 grown in nitrogen limited chemostat in two dilution rates. (b) Protein content analysis of the two strain.
